# Role of Phytoestrogen-Rich Bioactive Substances (*Linum usitatissimum* L., *Glycine max* L., *Trifolium pratense* L.) in Cardiovascular Disease Prevention in Postmenopausal Women: A Systematic Review and Meta-Analysis

**DOI:** 10.3390/nu14122467

**Published:** 2022-06-14

**Authors:** Agata Błaszczuk, Agnieszka Barańska, Wiesław Kanadys, Maria Malm, Monika Elżbieta Jach, Urszula Religioni, Rafał Wróbel, Jolanta Herda, Małgorzata Polz-Dacewicz

**Affiliations:** 1 Department of Virology with SARS Laboratory, Medical University of Lublin, 20-093 Lublin, Poland; agata.blaszczuk@umlub.pl (A.B.); malgorzata.polz.dacewicz@umlub.pl (M.P.-D.); 2 Department of Medical Informatics and Statistics with e-Health Lab, Medical University of Lublin, 20-090 Lublin, Poland; mariamalm@umlub.pl; 3 Specialistic Medical Center Czechow, 20-848 Lublin, Poland; wieslaw.kanadys@wp.pl; 4 Department of Molecular Biology, Faculty of Science and Health, John Paul II Catholic University of Lublin, 20-708 Lublin, Poland; monijach@kul.lublin.pl; 5 School of Public Health, Centre of Postgraduate Medical Education of Warsaw, 01-826 Warsaw, Poland; urszula.religioni@gmail.com; 6 Department of Developmental Dentistry, Medical University of Lublin, 20-081 Lublin, Poland; rafal.wrobel@umlub.pl; 7 Department of Public Health, Medical University of Lublin, 20-090 Lublin, Poland; jolantaherda@umlub.pl

**Keywords:** flaxseed, soy, red clover, lipid profile, meta-analysis, cardiovascular disease, botanical supplements, postmenopausal woman

## Abstract

The aim of this report was to determine the impact of flaxseed, soy and red clover, and their bioactive substances on the lipid profile in postmenopausal women in cardiovascular diseases prevention. We used the following databases: MEDLINE (PubMed), EMBASE and the Cochrane Library. Meta-analysis indicates that the intake of flaxseed by postmenopausal women is associated with a statistically significant reduction in total cholesterol (TC) levels (weighted-mean difference (WMD) = −0.26; 95% confidence interval (95% CI): −0.38 to −0.13; *p* = 0.0001), low-density lipoprotein cholesterol (LDL-C) levels (WMD = −0.19; 95% CI: −0.30 to −0.08; *p* = 0.0006), and high-density lipoprotein cholesterol (HDL-C) levels (WMD = −0.06; 95% CI: −0.11 to −0.01; *p* = 0.0150). The effect of soy protein on the lipid profile showed a significant decrease in TC levels: WMD = −0.15; 95% CI: −0.25–0.05; *p* = 0.0048, LDL-C levels: WMD = −0.15; 95% CI: −0.25–0.05; *p* = 0.0067, as well as a significant increase in HDL-C levels: WMD = 0.05; 95% CI: 0.02–0.08; *p* = 0.0034. Changes in the lipid profile showed a significant reduction in TC levels after the use of red clover (WMD = −0.11; 95% CI: −0.18–−0.04; *p* = 0.0017) and a significant increase in HDL-C levels (WMD = 0.04; 95% CI: 0.01 to 0.07; *p* = 0.0165). This meta-analysis provides evidence that consuming flaxseed, soy and red clover can have a beneficial effect on lipids in postmenopausal women and suggest a favorable effect in preventing cardiovascular diseases.

## 1. Introduction

Cardiovascular disease (CVD) is collection of disorders affecting the vasculature of the heart, brain and peripheral tissues, and remains the leading cause of death globally [1,2]. The most common cause of CV is atherosclerosis, which is initiated by an inflammatory reaction of the vascular endothelium [3]. The origins of these endothelial lesions are still not fully explained, but involved factors include: chronic elevations in blood pressure [4]; prolonged hyperglycemia and the resulting formation of advanced glycation end-products [5]; elevated lipoproteins, particularly molecules that have undergone oxidized modification [6]; and oxidative stress and inflammation [7]. With aging, a number of changes occur in the metabolism, known as the ‘metabolic syndrome’ [8]. Among others, these include the accumulation of fat mass in the abdominal compartment, transition to a more atherogenic lipid profile, hyperinsulinemia, insulin resistance and glucose intolerance [9,10]. The consequence of these changes is an enhanced risk of coronary heart disease, stroke and other atherosclerotic vascular diseases, including peripheral arterial disease, atherosclerotic aortic disease and carotid artery disease [11].

A bioactive effect on lipid metabolism involving lowering the level of total cholesterol (TC), low-density lipoprotein cholesterol (LDL-C) and triglycerides (TG), has been demonstrated during studies of some plant dietary items, such as: almonds [12], artichokes [13], barberry [14], curcumin [15], ginger [16], psyllium [17], sesame [18], cacao [19] and walnuts [20].

Women are at a higher risk of developing CVDs after menopause due to estrogen deficiency and dysregulated lipid metabolism [21]. Loss of ovarian endocrine function as a result of chronic hypoestrogenism is the main physiological symptom associated with menopause. The daily production of estrogen in postmenopausal women is 0.045 mg, compared with 0.35 mg during the reproductive period, which is reflected in serum estrogen concentrations of 10–20 µg/mL and 40–400 µg/mL, respectively [22]. Observed menopause-induced estrogen deficiency leads to various metabolic disorders including lipid metabolism. TC, LDL-C, and TG levels increase during the menopause and during the postmenopausal period. In turn, high-density lipoprotein cholesterol (HDL-C) levels, after an initial rise during the menopausal transition, gradually decline during late menopause [23,24,25] (of note, there were also studies showing no difference in HDL-C levels between premenopausal and postmenopausal women [26]). Dyslipidemia is one of the most important risk factors for CVD, which can be corrected and prevented. Botanical supplements as flaxseed, soybean and red clover are rich sources of bioactive compounds affecting lipid metabolism [27].

The benefits of consuming whole fractions of flaxseed (*Linum usitatissimum* L.) such as its protein, oil and mucilage, are related to the presence of specific bioactive substances. The flaxseed content of protein ranges from 10 to 31%, including higher amounts of arginine, aspartic and glutamic acids than other amino acids. Flaxseed also consists of 40% fat; and 25–28% fiber, of which 25% is in soluble form. Moreover, approximately 38–45% of flaxseed mass contains oil and 55–68% is meal. Flaxseed is a rich source of bioactive ingredients such as α-linolenic acid (ALA) and linoleic acid. Additionally, it contains phytochemicals such as lignan complex: secoisolariciresinol diglucoside (SDG), cinnamic acid glucoside and hydroxymethyl glutaric acid [28,29]. Flaxseed oil and active compounds, especially SDG and its metabolites, suppresses the inflammatory tissue damage caused by oxidative stress [30]. SDG may also directly lower serum cholesterol by modulating the enzymes 7α-hydroxylase and acyl-coenzyme A:cholesterol acyltransferases, both of which are involved in cholesterol metabolism [31]. The supplied ALA reduces the production of arachidonic acid (AA) and consequently, by decreasing proinflammatory eicosanoid, leads to a reduction in the inflammation process [32].

The soybean (*Glycine max* L.) is a significant source of protein (~36–40%), lipids (~20%) and dietary fiber (~9%) (based on the dry weight of mature raw seeds), and phytochemicals such as isoflavones, phytosterols and lecithins, which may act collectively or through independent mechanisms. The two major protein peptides, β-conglycinin (βCG) and glycinin, comprise 80–90% of the total protein in soybean, and affect lipid metabolism [33,34]. Additionally, soybeans are rich sources of essential fatty acids. Polyunsaturated (primarily linoleic acid, alpha-linolenic acid), monounsaturated (oleic acid) and saturated (primarily palmitic acid) fatty acids comprise approximately 63%, 23%, and 14%, respectively, of the total fat content of soybeans, and have an impact on the level of lipids [35]. The other major bioactive compounds in soybeans are isoflavones, which are associated with soy proteins. Isoflavones occur in large values in soybean as glycoside, such as genistin, daidzin and glycitin, or their aglycone forms, genistein, diadzein and glycitein [36]. Soy isoflavones, with structural similarities to the endogenous 17β-estradiol, reveal their biological effects via activating estrogen receptors (ER) with a higher affinity to ER-β, in comparison to ER-α. Although the affinity for the estrogen receptor by soy isoflavones is 100–1000 times less than that of natural estrogen, more than a thousand-fold greater isoflavone concentrations can appear in the plasma than those of endogenous estrogen [37]. Isoflavones, by binding to ERs, lead to gene activation and beneficial effects on lipid metabolism [38].

A number of other mechanisms regulating lipid metabolism without the mediation of the estrogen receptor have been recorded—including the increased expression of 3-hydroxy−3-methylglutaryl-CoA reductase (HMGCR), which leads to decreased cholesterol and TG levels; the enhanced expression of peroxisome proliferator-activated receptor (PPAR) and the activation of AMP-activated protein kinase (AMPK), which results in increased expression of genes involved in lipoprotein metabolism; the decreased expression of sterol regulatory-element binding protein-lc (SREBP-1) and increased expression of SREBP-2, which suppresses cholesterol synthesis and absorption in the liver; the inhibition of the expression and activity of the sterol regulatory element binding protein-1c (SREBP-1c) and carbohydrate response element binding protein-1 (ChREBP), which are proteins that enhance the expression of lipogenic genes and key enzymes involved in de novo lipogenesis; the promotion of the HDL-C metabolism and of the uptake, utilization and catabolism of fatty acids; and the modulation of the effects on several enzymes important in lipid transformation, such as lipoprotein lipase (LPL), hepatic lipase (HL) (also called hepatic triglyceride lipase (HTGL)), and 7alpha-hydroxylase [39,40,41,42,43,44].

Red clover (*Trifolium pratense* L.) contains a certain amount of protein and fat that is irrelevant from the point of view of human nutrition. It is also rich in bioactive substancesused in medicine. Red clover isoflavones show a different mechanism of action on lipid metabolism than that of soy isoflavones, which is due to the different composition of the contained isoflavones. Grains of red clover contain higher concentrations of formononetin and biochanin A and lower concentrations of daidzein and genistein than soy [45]. This composition suggests that an equal production status may be less relevant [46]. Isoflavones with structural similarities to endogenous 17-β-estradiol reveal their biological effects via activating estrogen receptors (ER) with a higher affinity to ER-β, in comparison to ER-α, which mediates the cholesterol metabolism [47,48]. In addition, a number of non-hormonal effects have been reported in its isoflavones, including tyrosine kinase inhibition, antioxidant activity, and effects on ion transport [49]. Red clover extract and the isoflavones genistein and biochanin A can also regulate lipid metabolism without the mediation of estrogen receptors, as well as increase the expression of PPAR alpha and activate AMPK, which results in the enhanced activity of genes involved in lipoprotein metabolism [50].

The purpose of this study was to determine the impact of flaxseed, soy and red clover and their bioactive substanceson the lipid profile in postmenopausal women in cardiovascular prevention.

## 2. Materials and Methods

### 2.1. Search Strategy and Study Selection

This systematic review and meta-analysis was designed in accordance with The Preferred Reporting Items for Systematic Reviews and Meta-analysis (PRISMA) statement [51] to identify randomized controlled trials (RCTs) assessing the effects of flaxseed, soy protein, soy isoflavones and red clover isoflavones on the level of serum lipids.

The electronic databases MEDLINE (PubMed), Embase, and the Cochrane Library were searched for the identification of randomized controlled trials until December 2018. The following search terms were used for all databases in various combinations: (“flax” OR “flaxseed” OR “linseed” OR “Linum usitatissimum” OR “soybean” OR “Glycine max” OR “soy proteins” OR “soy isoflavones” OR “red clover” OR “Trifolium pratense”) AND (“lipid profile” OR “lipids” OR “total cholesterol” OR “HDL cholesterol” OR “LDL cholesterol” OR “triglycerides”) AND (“menopause” OR “postmenopause”).

The search was limited to papers published in English and was conducted up to December 2018. References to selected research and review articles related to the topic of the work were also searched in order to identify additional studies.

The initial selection included the analysis of the titles and/or abstracts of all citations. After an independent and double analysis of the full texts of selected works, a decision was then made to include or exclude them. In turn, works were qualified for meta-analysis and collection of data on the clinical and methodological characteristics of the described clinical trials and for statistical evaluation.

Randomized controlled trials (RCTs) were considered eligible for inclusion if they met all of the following criteria: parallel-group design, or crossover design that contained data for the first period; a comparison with a placebo or with a no-intervention group; a follow-up period was at least 3 months; post-menopausal women as participants; appropriate interventions using flaxseed, soy or red clover and the presentation of sufficient information on plasma-lipid levels at baseline and after supplementation, or the net change values in both study arms. The exclusion criteria were as follows: men or premenopausal women as participants, no control group in the study, lack of sufficient information, and a study duration of less than 12 weeks. The results were reported as graphics or percent changes, and as duplicated reports.

### 2.2. Data Extraction

The data were extracted by the lead author and subsequently reviewed by co-authors for accuracy. Eligible studies were reviewed and the following data were abstracted: first author’s name; year of publication; study location (country); follow-up period of the study; study design; number of participants in the intervention and control group; health characteristics of the population (age, menopausal status, body mass index); daily amount of flaxseed, soy protein, soy isoflavones and red clover isoflavones taken in the active arm; and data on baseline and follow-up TC, LDL-C, HDL-C and TG plasma levels.

### 2.3. Quality Assessment and Bias Risk of the Trials

The Jadad Scale is an Oxford system for assessing the quality of a clinical trial, designed to determine the minimum level of studies included in a systematic review/meta-analysis. The test may receive values from 0 (low quality) to 5 points (highest quality) [52]. This meta-analysis included studies that had a relatively high Jadad score. To explain the possible presence of bias publications, Begg’s rank correlation test (Kendall Tau) and Egger’s weighted regression test were applied [53,54].

### 2.4. Statistical Analysis and Meta-Analysis

The meta-analysis included all intervention groups from multi-arm studies. Moreover, to avoid the duplication of data from the same people in surveys covering multiple time points, only one such point was taken into account.

The data in each study were presented as numbers of subjects (N) and the mean ± standard deviations (SD). When the standard error of the mean (SEM) was employed, the conversion to SD was made according to the formula: SD = SEM × √N. If a 95% confidence interval (95% CI) was applied, SD conversion was: SD = sqrt (N) × (upper bound–lower bound)/(2u) (equal to 3.96). When the results from the studies were presented in mg/dL, they were converted into mmol/L using standard conversion factors (the value in mg/dL was multiplied by 0.02586 for TC, LDL-C and HDL-C, and by 0.01143 for TG).

The outcome measures were the differences in the mean (MD) of components of the lipid profile between baseline and the end values for both the intervention and control groups. The missing SDs of MD were imputed using the formula: SD = sqrt ((SD “initial”)2 + (SD “final”)2 − (SD “initial” × SD “final”) × 2R), where R is the correlation coefficient; we took an R value = 0.40 [55,56]. The outcome measures were the differences in the mean (net change in mmol/L) of elements of the lipid profile between the baseline and the end values for both the intervention and control groups.

Summary outcomes measures were presented as the mean differences between the intervention and control groups. A random-effects model was used to calculate the weighted-mean difference (WMD) and 95% confidence interval (CI) for each comparison, and the combined overall effect (*p* < 0.05 was considered statistically significant) according to DerSimonian and Laird [57]. Cochrane Q and I^2^ statistics were used to assess the heterogeneity. The I^2^ test determined whether the variance across studies was correct and not a result of a sampling error. The percentage of total variation indicated the degree of heterogeneity; I^2^ values of ≤25% were considered low; >25% as moderate; and ≥75% as high heterogeneity [58]. STATISTICA Medical Software v. 11.0 StatSoft, Krakow, Poland was used for all statistical analyses.

## 3. Results

**Figure 1:**
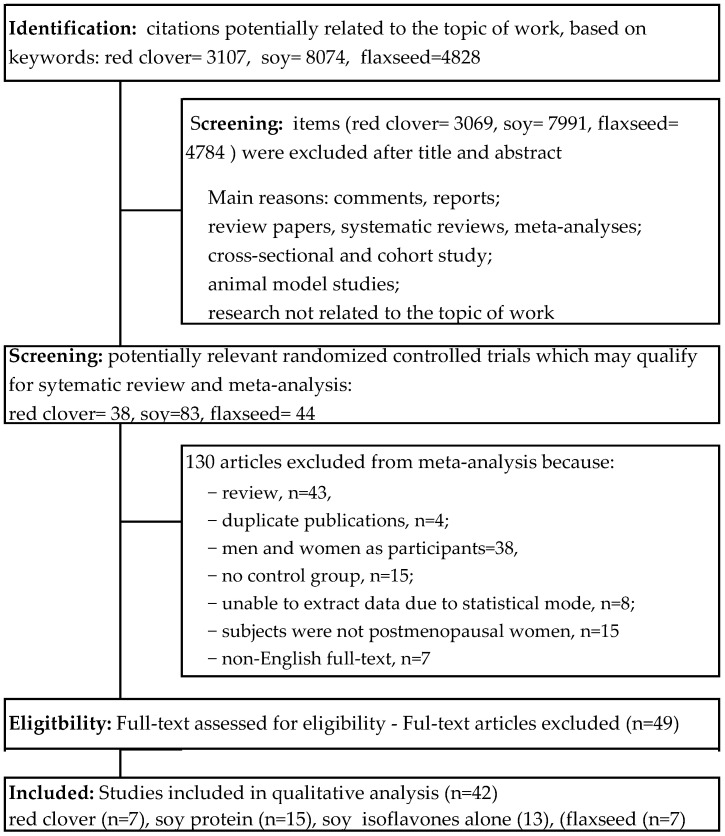
Flowchart of the selection procedure for studies included in the current review and meta-analysis.

In total, a number of citations potentially related to the topic of work based on the key words—red clover = 3107; soy = 8074; and flaxseed = 4828—were identified. Building upon the title and/or abstract, exclusions were 3069 for red clover; 7991 for soy; and 4784 for flaxseed due to a lack of connection with the topic of this work. Consequently, 165 potentially relevant clinical trials qualified for further detailed qualitative analysis in the full-text assessment: red clover = 38; soy = 83; and flaxseed = 44. Among these, 130 studies were also discarded due to the failure to meet all inclusion criteria. As a result, 42 randomized controlled trials for meta-analysis. Detailed information about the literature search and study selection and identification can be found in Figure 1.

### 3.1. Characteristics of Included Trials

The characteristics of selected randomized controlled studies assessing the influence of flaxseed, soy protein, soy isoflavones, and red clover on lipid profile in postmenopausal women are presented in Table 1. The meta-analysis included 42 studies published in English from 1998 to 2018 [59,60,61,62,63,64,65,66,67,68,69,70,71,72,73,74,75,76,77,78,79,80,81,82,83,84,85,86,87,88,89,90,91,92,93,94,95,96,97,98,99,100].

### 3.2. Associations between Flaxseed and Plasma Lipid Profiles

**Figure 2:**
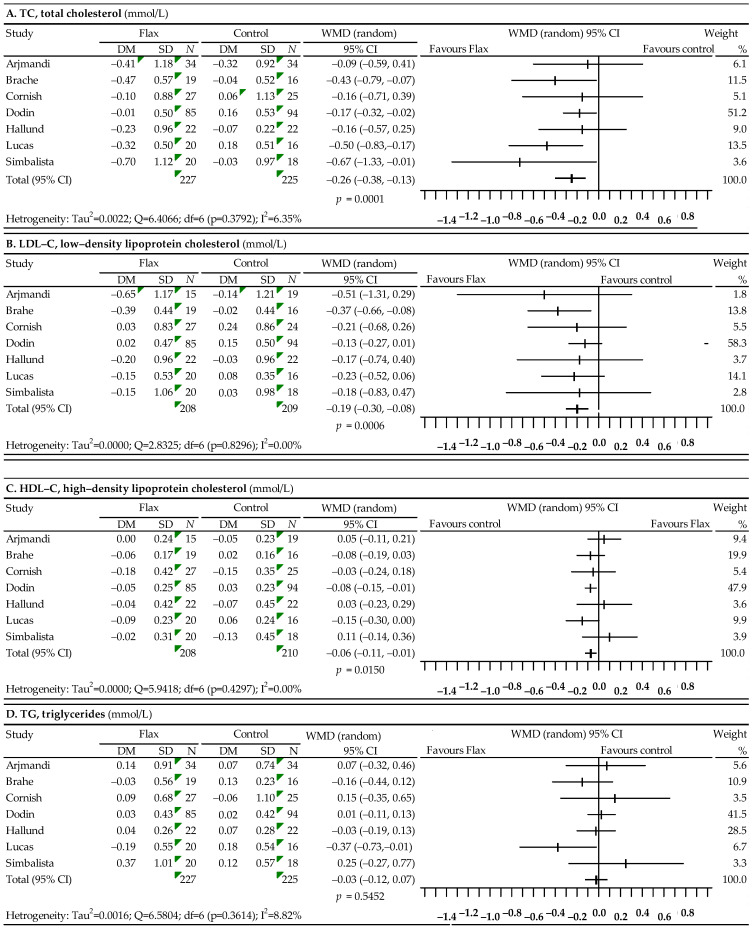
Forest plot representing the associations between flaxseed and lipid profiles. Data are presented as weighted mean difference with 95% CI.

Changes in lipid profile after the use of flaxseed were analyzed on the basis of seven studies [59,60,61,62,63,64,65]. The results of the meta-analysis are presented in Figure 2. Compared to the control group, the use of flaxseed resulted in a statistically significant reduction in TC levels (WMD = −0.26; 95% CI: −0.38–−0.13; *p* = 0.0001), LDL-C levels (WMD = −0.19; 95% CI: −0.30–−0.08; *p* = 0.0006) and HDL-C levels (WMD = −0.06; 95% CI: −0.11–−0.01; *p* = 0.0150) and a slight, not statistically significant reduction in TG levels: WMD = −0.03; 95% CI: −0.12–0.07; *p* = 0.5452. The heterogeneity analysis performed for TC, LDL-C, HDL-C and TG did not show that the differences between the effects obtained in different studies were statistically significant. The Begg and Egger asymmetry tests showed no publication bias for TC (*p*-value 0.6523 and 0.3091, respectively), LDL-C (*p*-value 0.6523 and 0.1786, respectively), HDL-C (*p*-value 0.1765 and 0.1578, respectively) or TG (*p*-value 0.4527 and 0.9335, respectively).

### 3.3. Associations between Soy Protein without and with Isoflavones and Lipid Profiles

**Figure 3:**
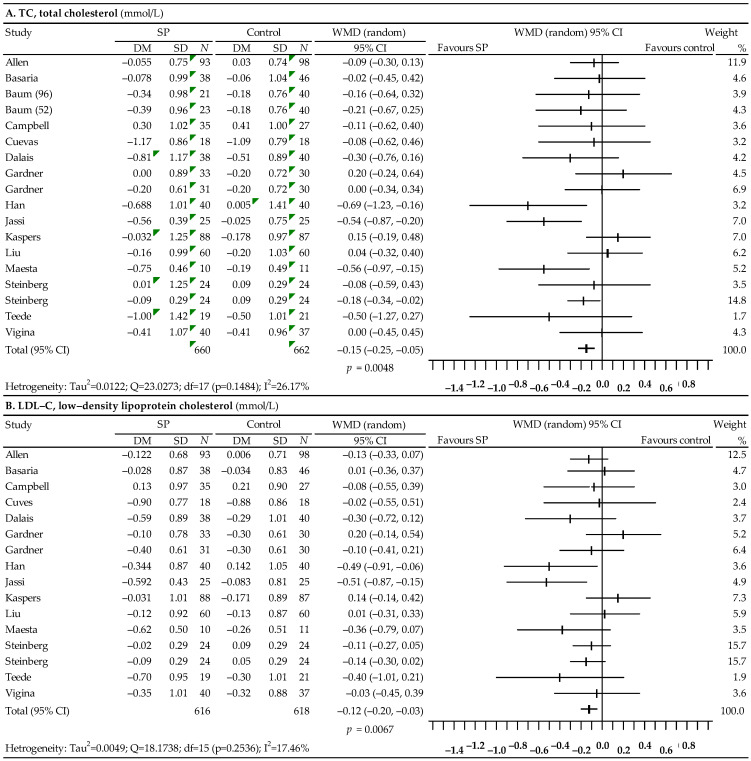

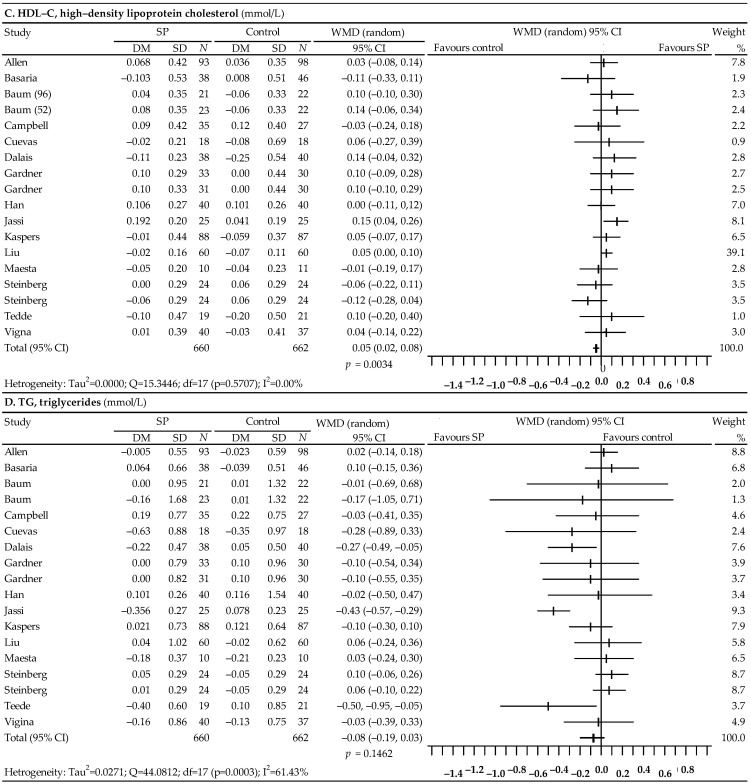
Forest plot representing the associations between soy protein and lipid profiles. Data are presented as weighted mean difference with 95% CI.

Fifteen studies were used in the analysis of the effect of soy protein on the lipid profile [66,67,68,69,70,71,72,73,74,75,76,77,78,79,80], but the data from the study by Baum et al. did not allow for a comparison of the effect in the case of LDL-C levels [68]. The results of the meta-analysis are presented in Figure 3. Statistical analysis showed a significant decrease in TC levels: WMD = −0.15; 95% CI: −0.25–0.05; *p* = 0.0048, LDL-C levels: WMD = −0.15; 95% CI: −0.25–0.05; *p* = 0.0067, and a significant increase in HDL-C levels: WMD = 0.05; 95% CI: 0.02–0.08; *p* = 0.0034. There was also a slight reduction in TG levels, which, however, was statistically non-significant (WMD = −0.08; 95% CI: −0.19 to 0.03; *p* = 0.1462). The performed analysis of heterogeneity did not show statistically significant differences between the effects of the included studies for TC, LDL-C and HDL-C, but in the case of TG, the heterogeneity was high (I^2^ = 61.43%). Begg’s test gave a statistically non-significant result for TC (*p* = 0.2403), as well as LDL-C (*p* = 0.4421), HDL-C (*p* = 0.8196) and TG (*p* = 0.0945), which indicated no publication bias. Moreover, Egger’s test showed no publication bias for TC: *p* = 0.6815, LDL-C: *p* = 0.5596, HDL-C: *p* = 0.6843, and TG: *p* = 0.8158.

### 3.4. Associations between Soy Isoflavones Alone (Preparation) and Lipid Profiles

**Figure 4:**
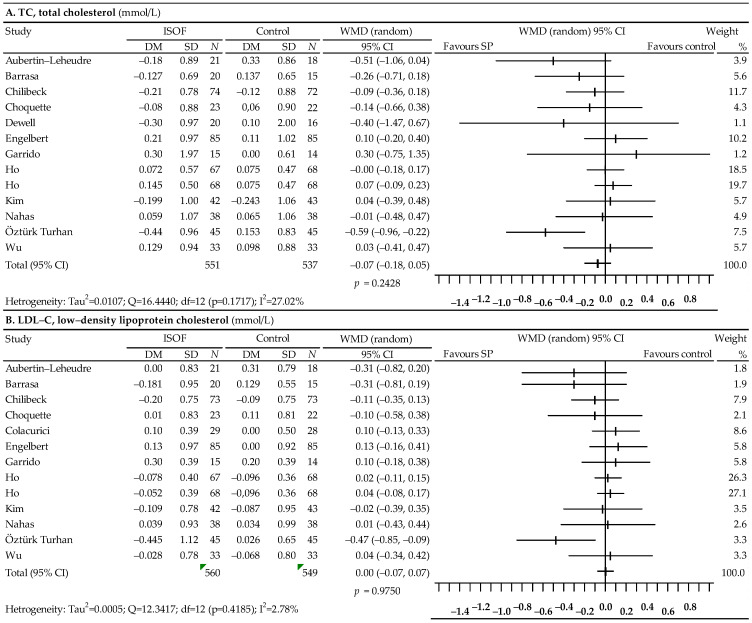

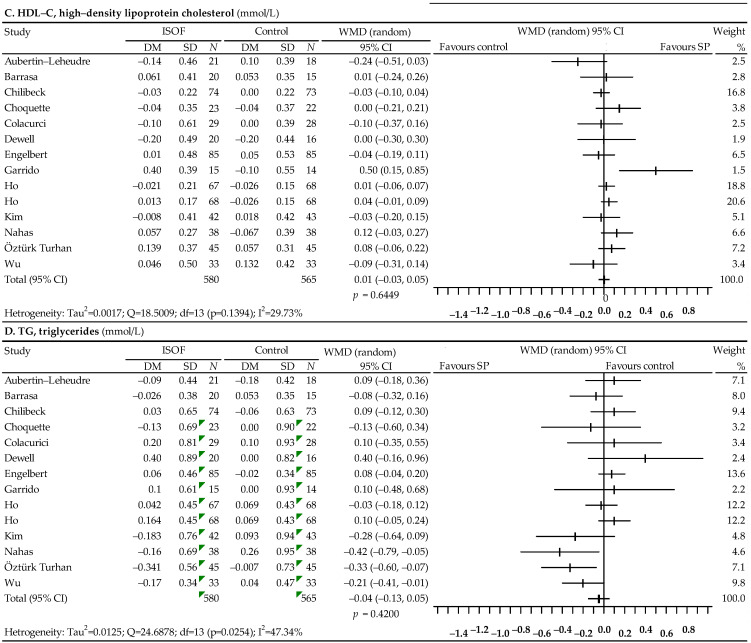
Forest plot representing associations between isoflavones and lipid profiles. Data are presented as the weighted mean difference with 95% CI.

A total of 13 studies were selected to analyze the effect of soy isoflavones on the lipid profile [81,82,83,84,85,86,87,88,89,90,91,92], among which the data from the Colacurici et al. [93] did not allow for the analysis of the effect of isoflavones on TC, while in the study by Dewell et al. [85], there were insufficient data on LDL-C. The results of the meta-analysis are shown in Figure 4. A slight, statistically insignificant decrease in TC levels was observed: WMD = −0.07; 95% CI: −0.18–0.05; *p* = 0.2428, as well as TG: WMD = −0.04; 95% CI: −0.13–0.05; *p* = 0.4200. On the other hand, no effect of the use of isoflavones on LDL-C levels was noticed: WMD = 0.00; 95% CI: −0.07–0.07; *p* = 0.9750 and HDL-C: WMD = 0.01; 95% CI: −0.03–0.05; *p* = 0.6449. The heterogeneity of the studies was not significant in the case of TC, LDL-C and HDL-C, but it turned out to be high in the case of TG (I^2^ = 47.34%). The results for the asymmetry tests were not statistically significant for TC: Begg’s test—*p* = 0.0672; Egger’s test—*p* = 0.1619, LDL-C: Egger’s test—*p* = 0.0872, HDL-C: Begg’s test—*p* = 0.7016; Egger’s test—*p* = 0.9451 and TG: Begg’s test—*p* = 0.3520; Egger’s test—*p* = 0.3281. However, Begg’s test showed a statistically significant publication bias for LDL-C (*p* = 0.0281).

### 3.5. Associations between Red Clover and Lipid Profiles

**Figure 5:**
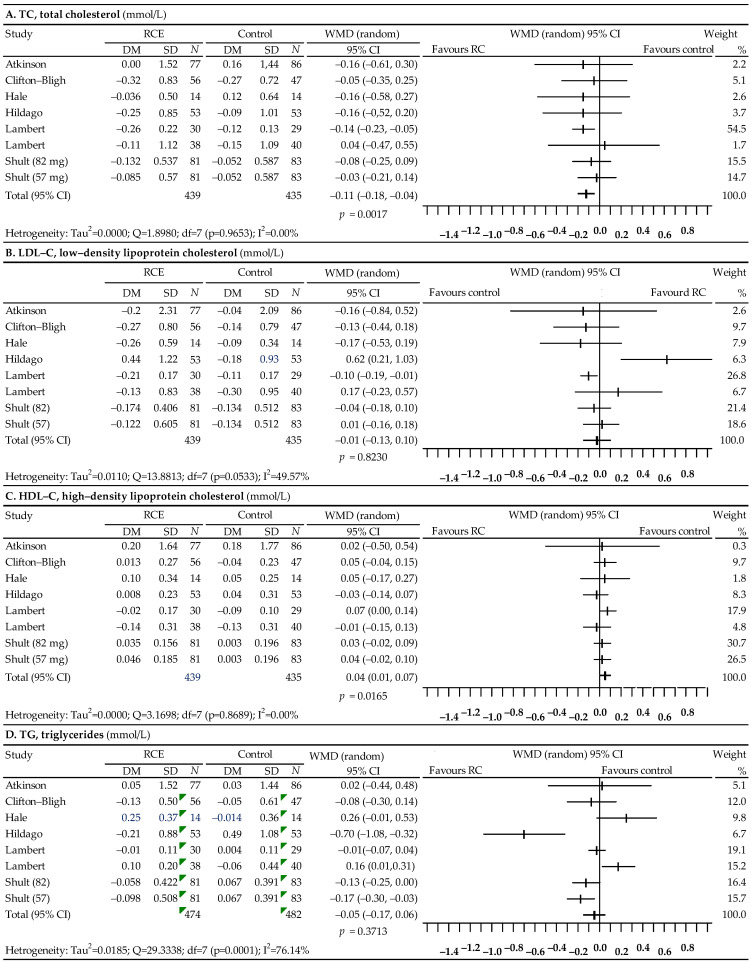
Forest plot representing associations between red clover and lipid profiles. Data are presented as weighted mean difference with 95% CI.

The last analysis, presented in Figure 5, concerned the effect of red clover on the lipid profile, and included seven studies [94,95,96,97,98,99,100]. There was a significant reduction in TC levels after the use of red clover (WMD = −0.11; 95% CI: −0.18–−0.04; *p* = 0.0017) and a statistically significant increase in HDL-C levels (WMD = 0.04; 95% CI: 0.01 to 0.07; *p* = 0.0165). In the case of TC and HDL-C, no significant heterogeneity of the study effects was observed, and publication bias was not demonstrated. The *p* value of Begg’s test was 0.4579 for TC and 0.6207 for HDL-C, while the *p* value of Egger’s test was 0.3990 for TC and 0.5319 for HDL-C. In contrast, statistical analysis showed no significant changes in LDL-C levels after the use of red clover (WMD = −0.01; 95% CI: −0.13 to 0.10; *p* = 0.8230) and showed a slight decrease in TG levels, which was statistically insignificant (WMD = −0.05; 95% CI: −0.17–0.06; *p* = 0.3713). In the case of LDL-C and TG, the heterogeneity of the studies turned out to be high (I^2^ = 49.57% and I^2^ = 76.14%, respectively). The asymmetry tests showed no publication bias. The *p* value of Begg’s test was 0.4527 for LDL-C and 0.4527 for TG, while the *p* value of Egger’s test was 0.2560 for LDL-C and 0.6425 for TG.

## 4. Discussion

The present meta-analysis indicates that the intake of flaxseed by postmenopausal women is associated with a statistically significant reduction in TC levels (WMD = −0.26; 95% CI: −0.38 to −0.13; *p* = 0.0001), LDL-C levels (WMD = −0.19; 95% CI: −0.30 to−0.08; *p* = 0.0006), HDL-C levels (WMD = −0.06; 95% CI: −0.11 to −0.01; *p* = 0.0150). These findings are consistent with previous published meta-analyses for the flaxseed effect. A meta-analysis by Hadi et al. incorporating 62 randomized trials involving dietary supplementation with flaxseed or flaxseed-derived products showed that flaxseed supplementation significantly reduced TC (WMD = −5.389 mg/dL; 95% CI: −9.483, −1.295, *p* = 0.010), TG (WMD = −9.422 mg/dL; 95% CI: −15.514, −3.330, *p* = 0.002), and LDL-C (WMD = −4.206 mg/dL; 95% CI: −7.260, −1.151, *p* = 0.007) concentrations. However, it had no effect on HDL-C (WMD = 0.047 mg/dL; 95% CI: −0.777, 0.872, *p* = 0.910) [101]. The meta-analysis of Yang et al. indicated that different flaxseed products showed different effects. Whole flaxseed supplementation significantly reduced TC (−11.85 mg/dL, 95% CI −20.12–−3.57, *p*  =  0.005), LDL-C (− 10.51 mg/dL, 95% CI −14.96–−6.06, *p* < 0.001), TG (−19.77 mg/dL, 95% CI −33.61–−5.94, *p*  =  0.005), TC/HDL-C (− 0.10, 95% CI −0.19–−0.003, *p*  =  0.044), while lignans supplementation significantly reduced TC (− 17.86 mg/dL, *p*  =  0.004), LDL-C (− 15.47 mg/dL, *p*  <  0.001), and TC/HDL-C (− 0.45, *p*  =  0.04). Flaxseed oil supplementation had no such lowering effect on lipid [102].

Our meta-analysis of the effect of soy protein on the lipid profile showed a significant decrease in TC levels: WMD = −0.15; 95% CI: −0.25–0.05; *p* = 0.0048, LDL-C levels: WMD = −0.15; 95% CI: −0.25–0.05; *p* = 0.0067, as well as a significant increase in HDL-C levels: WMD = 0.05; 95% from CI: 0.02 to 0.08; *p* = 0.0034. There was also a slight reduction in TG levels, which, however, was statistically non-significant (WMD = −0.08; 95% CI: from −0.19 to 0.03; *p* = 0.1462). The meta-analysis by Moradi et al. supports the hypercholesterolemic effect of soy lowering the serum TC levels. Soy consumption was associated with a significant decrease in TG: −5.04 mg/dL; 95% CI: −9.95, −0.13; *p* = 0.044), TC (MD: −3.02 mg/dL; 95% CI: −5.56, −0.47; *p*= 0.02), LDL-C (3.27 mg/dL; 95% CI: −6.01, −0.53; *p* = 0.019) and HDL-C (MD: −2.28 mg/dL; 95% CI: −4.27, −0.29; *p* = 0.025). The reductions in LDL-C, TG, and HDL-C were larger in subjects consuming isolated soy protein than taking-in isolated soy isoflavones [37]. The results of previous meta-analyses also revealed a significant decrease in serum TC, LDL-C, and TG concentrations after the consumption of soy protein containing isoflavones [103].

This meta-analysis showed a significant reduction in TC levels after the use of red clover (WMD = −0.11; 95% CI: from −0.18 to −0.04; *p* = 0.0017) and a significant increase in HDL-C levels (WMD = 0.04; 95% CI: from 0.01 to 0.07; *p* = 0.0165). However, the study demonstrated no significant changes in LDL-C levels (WMD = −0.01; 95% CI: from −0.13 to 0.10; *p* = 0.8230) and a slight statistically insignificant decrease in TG levels (WMD = −0.05; 95% CI: from −0.17 to 0.06; *p* = 0.3713) after the use of red clover. In their meta-analysis, Luis et al. verified that the consumption of red clover by perimenopausal and postmenopausal women results in a significant decrease in TC, LDL-C, and TG, together with a significant increase in HDL-C [104]. Furthermore, the meta-analysis by Kanadys et al. revealed changes in serum levels: TC, −0.29 (95 % CI: from −0.53 to −0.06) mmol/L, *p* = 0.0136; LDL-C, −0.13 (95 % CI: from −0.35 to 0.09) mmol/L, *p* = 0.2418; TG, −0.15 (95 % CI: from −0.32 to 0.01) mmol/L, *p* = 0.0592; and HDL-C, 0.14 (95 % CI: from −0.08 to 0.36) mmol/L, *p* = 0.2103—which suggest benefits from red clover consumption specific to correcting abnormal cholesterol levels [105].

### Study Limitations

Despite the results obtained in this systematic review and its meta-analysis, some limitations were found. Because of the lack of standardization in some of the study designs, such as the ingredients and doses of isoflavones and the durations and outcomes of the trials, it currently remains difficult to draw overall conclusions for all aspects of isoflavone intake. These limitations warrant further investigation with regard to the use of isoflavone in women’s health. Study limitations can be also be found due to individual differences in the bioavailability of individual components of preparations as these were prepared in a variety of ways that were suitable for each study. Moreover, limitations were posed by potential publication bias, which is revealed via the asymmetry of the funnel plot and the Egger’s model. Publication bias suggests that some small studies with negative findings may have been missed or unpublished. Additionally, effects on vascular function have hardly been studied and more studies are needed to better establish what the effect of flaxseed, soy, red clover are on heart and vascular function.

## 5. Conclusions

This meta-analysis provides evidence that consuming flaxseed, soy, and red clover can have a beneficial effect on lipids in postmenopausal women. Their consumption could provide an important strategy to control dyslipidemia, and therefore, natural products can be an alternative to medicaments for preventing CVD, which has some clinical relevance in anti-atherosclerotic therapy. Our data also suggest that future well-designed studies with large sample sizes and adequate durations are needed to fully investigate the effectiveness of flaxseed, soy, and red clover.

## Figures and Tables

**Table 1:** Characteristics of selected randomized controlled studies assessing the influence of flaxseed, soy protein, soy isoflavones, and red clover on lipid profile in postmenopausal women.

First Author [Ref.] Data Location	Study Design Trial Duration	Study Population Age (Mean ± SD) y, ysm, BMI, Health Condition	Intervention (Daily Dose)	GroUp Studied	Number Sample	Baseline Lipids Values				Jadad Score
						Total-C mmol/L	LDL-C mmol/L	HDL-C mmol/L	TAG mmol/L	
A. Flaxseed (*Linum usitatissimum* L.)										
Arjmandi [59] 1998 United States	Cross-over 6-week active phase 2-week washout.	Age 56.3 ± 6.5, ysm N/A, BMI 29.2 ± 7.4, obesity, hypercholesterolemia	WFX 38 g, ALA 8.5 g vs. placebo: sunflower seed (slice of bread or muffin)	FG CG	15 19	5.95 ± 1.44 5.92 ± 1.36	4.12 ± 1.39 4.06 ± 1.34	0.93 ± 0.23 1.08 ± 0.23	1.28 ± 0.92 1.27 ± 0.70	4
Lucas [64] 2002 United States	Parallel group 3-month follow-up	Age 54 ± 8, ysm N/A, BMI 29.1 ± 7.1 obesity	WFX 40 g vs. placebo, wheat-based 40 g	FG CG	20 16	5.76 ± 1.12 5.95 ± 1.12	3.21 ± 1.12 3.52 ± 1.12	1.89 ± 0.42 1.61 ± 0.40	1.48 ± 0.71 1.56 ± 0.76	4
Dodin [62] 2005 Canada	Parallel group 1-year follow-up	Age 54.0 ± 4.0, ysm 4.7 ± 5.2, BMI 25.5 ± 4.5 healthy	WFX 40 g, ALA 9.1 g vs. control, wheat germ (slice of bread or drinks)	FG CG	85 94	5.67 ± 0.75 5.78 ± 0.71	3.43 ± 0.69 3.50 ± 0.64	1.72 ± 0.33 1.74 ± 0.39	1.12 ± 0.45 1.16 ± 0.57	5
Hallund [63] 2006 Denmark	Cross-over 6-week active phase 6-week washout	Age 61 ± 7, ysm >24 mo, BMI 25.5 ± 4.5 healthy	Lignan complex, SDG 500 mg vs. control (in form muffins, 50 g)	FG CG	22 22	6.05 ± 1.03 6.03 ± 0.98	3.80 ± 1.03 3.79 ± 0.98	1.81 ± 0.42 1.82 ± 0.52	0.96 ± 0.28 0.93 ± 0.33	4
Cornish [61] 2009 Canada	Parallel group 6-month follow-up	Age 59.7 ± 5.3, ysm N/A, BMI 27.1 ± 5.3 healthy	Lignan complex, SGD 500 mg vs. placebo	FG CG	27 25	5.87 ± 0.88 6.14 ± 1.05	3.60 ± 0.88 3.77 ± 0.80	1.74 ± 0.42 1.54 ± 0.40	1.19 ± 0.68 1.77 ± 1.10	4
Simbalista [65] 2010 Brazil	Parallel group 3-month follow-up	Age 52.0 ± 2.9, ysm 3.8 ± 2.3, BMI 26 ± 3.6, healthy	GFX: WFX 25 g, SDG 46 mg, vs placebo: wheat bran (in form of slice bread)	FG CG	20 18	6.03 ± 0.87 5.18 ± 0.93	3.83 ± 0.89 2.87 ± 0.93	1.61 ± 0.31 1.86 ± 0.42	1.49 ± 0.80 1.00 ± 0.54	5
Brache [60] 2015 Denmark	Parallel group 6-week follow-up	Age 60.6 ± 6.4 y, ysm ≥1 y, BMI 35.2 ± 4.5, obesity	10 g flaxseed mucilage vs. placebo: maltodextrin (in form buns)	FG CG	19 16	6.39 ± 0.89 5.76 ± 0.69	4.11 ± 0.84 3.44 ± 0.74	1.40 ± 0.22 1.56 ± 0.42	1.51 ± 0.77 1.07 ± 0.32	3
**B. Soybean (*Glycine max* (L.) Merr.)**										
**B. 1. Soy protein without and with isoflavones**										
Baum [68] 1998 United States	Parallel groups 2-week run-in/ 12-week follow-up	Age 60.8 ± 8.6 y, ysm N/A, BMI 27.8 ± 5.3, hypercholesterolemia	a. SP 40 g: a. IAE 90 mg; b. SP 40 g; IAE 56 mg vs. control, CP + MP 40 g	SG 90 SG 56 CG	21 23 22	6.47 ± 0.88 6.57 ± 0.85 6.26 ± 0.67	N/A N/A 4.9 ± 0.8	1.38 ± 0.32 1.34 ± 0.28 1.38 ± 0.31	1.74 ± 0.75 1.89 ± 1.02 1.75 ± 1.11	3
Vigna [80] 2000 Italy	Parallel groups 12-week follow-up	Age 53.4 ± 3.3, ysm 2.4 y, BMI 25.9 ± 3.5, healthy	SP 40 g, IF 76 mg vs. control, CP 40 g	SG CG	40 37	6.37 ± 1.01 6.55 ± 0.93	4.13 ± 0.87 4.33 ± 0.87	1.57 ± 0.36 1.61 ± 0.38	1.47 ± 0.90 1.32 ± 0.77	4
Gardner [72] 2001 United States	Parallel groups 4-week run-in/ 12-week follow-up	Age 59.9 ± 6.6, ysm N/A, BMI 26.3 ± 4.6, hypercholesterolemia	a. SP 42 g b. SP 42 g (52 mg Gen, 25 mg Dai, 4 mg Gly) vs. control, MP 42 g.	SG SG CG	33 31 30	5.9 ± 0.7 5.9 ± 0.6 6.1 ± 0.6	3.9 ± 0.6 3.9 ± 0.6 4.0 ± 0.5	1.4 ± 0.3 1.5 ± 0.3 1.5 ± 0.4	1.3 ± 0.5 1.3 ± 0.8 1.3 ± 0.7	4
Han [73] 2002 Brazil	Parallel groups 4-month follow-up	Age 48.5 ± 7.6, ysm 1.9 ± 1.6 y, BMI 24.3 ± 3.2, healthy	SP 50.3 mg, IAE 23.3 mg Gen, 3.8 mg Gly, 6.2 mg Dai) vs. placebo	SG CG	40 40	5.83 ± 0.88 5.86 ± 1.26	3.45 ± 0.87 3.45 ± 1.32	1.04 ± 0.23 1.03 ± 0.21	2.31 ± 1.66 1.99 ± 1.66	5
Dalais [71] 2003 Australia	Parallel groups 3-month follow-up	Age 60 ± 6.2, ysm N/A, BMI 25.3 ± 4.6, healthy	SP 40 g, IC 118 mg (69 mg Agl) vs. control, CP 40 g	SG CG	38 40	6.12 ± 0.92 5.92 ± 0.88	4.00 ± 0.86 3.69 ± 0.88	1.63 ± 0.49 1.72 ± 0.51	1.09 ± 0.68 1.01 ± 0.57	5
Steinberg [78] 2003 United States	Cross-over 6-week active phase 4-week washout	Age 5.49 ± 5.29, ysm N/A, BMI 24.6 ± 3.2, healthy	a. SP 25 g b. SP 25 g, IAE 107 mg (55 mg Gen, 47 mg Dai, 5 mg Gly) vs. control, MP 25 g	SG a SG b CG	24 24 24	4.91 ± 0.49 4.91 ± 0.49 4.91 ± 0.49	2.89 ± 0.49 2.89 ± 0.49 2.89 ± 0.49	1.55 ± 0.49 1.55 ± 0.49 1.55 ± 0.49	1.03 ± 0.49 1.03 ± 0.49 1.03 ± 0.49	4
Cuevas [70] 2003 Chile	Cross-over 8-week active phase 4-week washout	Age 59 y, ysm 10 y, BMI 29.3 ± 3.43, obesity, hypercholesterolemia	SP 40 g, IAE 80 mg (60% Gen, 30% Dai, 10% Gly) vs. control, caseinate 40 g	SG CG	18 18	7.90 ± 0.74 7.90 ± 0.74	5.04 ± 0.66 5.04 ± 0.66	1.39 ± 0.27 1.39 ± 0.27	2.18 ± 0.83 2.18 ± 0.83	4
Kreijkamp- Kaspers [75] 2004 Netherlands	Parallel groups 12-month follow-up	Age 66.6 ± 4.7, ysm 17.9 ± 6.9 y, BMI 26.1 ± 3.8, healthy	SP 25.6 g, IAE 99 mg (52 mg Gen, 6 mg Gly, 41 mg Dai) vs. control, MP 25,6 mg	SG CG	88 87	6.21 ± 0.73 6.11 ± 0.95	4.16 ± 0.99 4.12 ± 0.88	1.55 ± 0.41 1.53 ± 0.34	1.36 ± 0.72 1.25 ± 0.59	4
Teede [79] 2005 Australia	Parallel groups 3-day run-in/ 3-month follow-up	Age 59.5 ± 4.5, ysm N/A, BMI 25.9 ± 5.4, healthy	SP 40 g, IC 118 mg (54 mg Gen, 3.6 mg Gly, 26 mg Dai) vs. control, CP 40 g	SG CG	19 21	6.2 ± 1.30 5.8 ± 0.92	4.0 ± 0.87 3.6 ± 0,92	1.6 ± 0.43 1.6 ± 0.46	1.0 ± 0.48 1.0 ± 0.63	3
Allen [66] 2007 United States	Parallel groups 4-week run-in/ 12-week follow-up	Age 56.8 ± 5.6, ysm 9.4 ± 8.3 y, BMI 27.9 ± 4.7, hypercholesterolemia	SP 20 g, IC 160 mg (~96 mg Agl) vs. control, MP 20 g	SG CG	93 98	5.80 ± 0.68 5.71 ± 0.64	3.67 ± 0.68 3.60 ± 0.57	1.56 ± 0,37 1.52 ± 0.31	1.25 ± 0.51 1.28 ± 0.60	5
Maesta [77] 2007 Brazil	Parallel group 16-week follow-up	Age 61.3 ± 5,2, ysm 10.7 ± 4.9 y, BMI 27.2 ± 5.3 healthy	SP 25 g, IAE 50 mg (32 mg Gen, 15 mg Dai, 3 mg Gly) vs. placebo, maltodextrine	SG CG	10 11	5.95 ± 0.71 5,76 ± 0.98	3.71 ± 0.72 3.56 ± 0.70	1.62 ± 0.34 1.32 ± 0.25	1.36 ± 0.52 1.95 ± 0.71	5
Basaria [67] 2009 United States	Parallel groups 12-week follow-up	Age 55.7 ± 1.3, ysm 5.7 ± 0.9, BMI 26.1 ± 0.8, healthy	SP 20 g, IC 160 mg (IAE: 64 mg Gen, 63 mg Dai, 34 mg Gly) vs. control, MP 20 g	SG CG	38 46	5.48 ± 0.14 5.69 ± 0.85	3.15 ± 0.75 3.21 ± 0.74	1.88 ± 0.46 2.02 0.46	1.03 ± 0.58 0.99 ± 0.46	4
Campbell [69] 2010 United States	Parallel groups 12-month follow-up	Age 54.7 ± 5.5, ysm 5.5 ± 5.0, BMI 27.9 ± 5.9, hypercholesterolemia	SP 25 g, 60 mg IF vs. control, CP 25 g	SG CG	35 27	5.97 ± 0,93 6.15 ± 0.91	3.88 ± 0.90 3.95 ± 0.87	1.47 ± 0.38 1.50 ± 0.36	1.34 ± 0.70 1.48 ± 0.67	4
Jassi [74] 2010 India	Parallel groups 12-week follow-up	Age 51.1 ± 8.6, ysm 2.3 ± 1.2, BMI 23.4 ± 2.7, healthy	SP 30 g, IF 60 mg vs. control, CP 30 g	SG CG	25 25	4.96 ± 0.36 4.69 ± 0.71	3.09 ± 0.37 2.83 ± 0.76	1.06 ± 0.15 1.06 ± 0.16	1.76 ± 0.28 1.76 ± 0.17	4
Liu [76] 2012 Hong Kong SAR	Parallel groups 2-week run-in/ 3-month follow-up	Age 56.3 ± 4.3, ysm 5.9 ± 5.4, BMI 24.4 ± 3.6, prediabetes	SP 15 g, IAE 100 mg (59 mg Gen,4 mg Gly, 35 mg Dai) vs. control, MP 15 g	SG CG	60 60	5.83 ± 0.94 5.63 ± 0.93	3.94 ± 0.67 3.81 ± 0.88	1.66 ± 0.31 1.65 ± 0.30	1.35 ± 1.19 1.30 ± 0.70	5
**B.2. Soy isoflavones preparations**										
Dewell [85] 2002 USA	Parallel groups 2-month follow-up	Age 69.5 ± 4.2 y, ysm N/A, BMI 25.0 ± 4,2, moderate hypercholesterolemia	IC 150 mg (90 mg Agl: 45 mg Gen, 55% Dai and Gly) vs. placebo	SG CG	20 16	6.8 ± 0.9 6.3 ± 2.0	N/A N/A	1.2 ± 0.5 1.2 ± 0.4	0.8 ± 0.5 1.3 ± 0.8	4
Colacurci [93] 2005 Italy	Parallel groups 6-month follow-up	Age 55.1 ± 38 y, ysm 4.9 ± 0.6, BMI 25.9 ± 1.8, healthy	IAE 60 mg (30 mg Gen, 30 mg Dai) vs. placebo	SG CG	29 28	NR NR	3.7 ± 0.3 3.6 ± 0.4	1.06 ± 0.5 1.05 ± 0.5	1.5 ± 0.6 1.6 ± 0.8	4
Garrido [87] 2006 Chile	Parallel groups 12-week follow-up	Age 55.5 ± 4.0 y, ysm N/A, BMI 26.9 ± 2.3, healthy	IAE ~100 mg (46.8 mg Gen, 48.2 mg Dai) vs. placebo	SG CG	15 14	5.5 ± 1.0 4.8 ± 0.5	3.4 ± 0.4 2.9 ± 03	1.4 ± 0.3 1.8 ± 0.6	1.3 ± 0.2 1.4 ± 0.2	3
Wu [92] 2006 Japan	Parallel group 6-month follow-up	Age 54.4 ± 2.9 y, ysm N/A, BMI 21.1 ± 2.4, healthy	IC 75 mg (47 mg Agl: 38.3 mg Dai, 8.6 mg, 1 mg Gly) vs. placebo	SG CG	25 29	5.90 ± 0.76 5.88 ± 0.86	3.52 ± 0.72 3.59 ± 0.76	1.92 ± 0.47 1.85 ± 0.38	0.95 ± 0.43 1.16 ± 0.53	3
Nahas [90] 2007 Brazil	Parallel groups 4-week run-in 4-month follow-up	Age 55.7 ± 6.8, ysm 6.9 ± 4.5, BMI 29.1 ± 5.0, obesity	IC 100 mg (50% Gen, 35% Dai), vs. placebo	SG CG	38 36	5.56 ± 0.92 5.37 ± 0.97	3.47 ± 0.82 3.26 ± 0.82	1.29 ± 0.27 1.35 ± 0.34	1.73 ± 0.74 1.67 ± 0.89	3
Ho [88] 2007 China	Parallel groups 6-month follow up	Age 54.2 ± 3.1, ysm 4,1 ± 2.4, BMI 24.1 ± 3.6, healthy	a. IAE 80 mg, b. IAE 40 mg (46.4% Dai, 38.8 Gly, 14.7% Gen) vs. placebo	SG 80 SG 40 CG	67 68 68	5.86 ± 0.83 5.83 ± 0.84 5.93 ± 0.89	3.19 ± 0.74 3.23 ± 0.68 3.25 ± 0.73	1.89 ± 0.41 1.80 ± 0.39 1.86 ± 0.42	1.13 ± 0.56 1.32 ± 0.93 1.29 ± 0.96	4
Aubertin-Leheudre [81] 2008 Canada	Parallel groups 6-month follow-up	Age 57.4 ± 5.4 y, ysm 8.6 ± 7.5, BMI 32.0 ± 12.5, obesity	IAE 70 mg (44 mg Dai, 16 mg Gly, 10 mg Gen) vs. placebo	SG CG	21 18	5.41 ± 0.88 5.33 ± 0.83	3.17 ± 0.81 3.17 ± 0.78	1.55 ± 0.49 1.45 ± 0.37	1.51 ± 0.69 1.52 ± 0.69	4
Özturk Turhan [91] 2009 Turkey	Parallel groups 6-month follow-up	Age 51.5 ± 5.1; ysm 3.6 ± 1.7, BMI 27.1 ± 3.1	IAE 40 mg (29.8 mg Gen, 7.8 mg Dai, 2.4 mg Gly) vs. placebo	SG CG	45 45	6.82 ± 0.96 6.30 ± 0.76	4.25 ± 0.73 4.01 ± 0.65	1.06 ± 0.15 1.06 ± 0.16	1.76 ± 0.28 1.76 ± 0.17	4
Choquette [84] 2011 Canada	Parallel groups 6-month follow-up	Age 58.5 ± 5.5 y, ysm 9.0 ± 7.0, BMI 30.1 ± 2.7, obesity	IAE 70 mg (44 mg Dai, 16 mg Gly, 10 mg Gen) vs. placebo	SG CG	23 22	5.40 ± 0.80 5.58 ± 0.86	3.34 ± 0.75 3.34 ± 0.81	1.49 ± 0.34 1.37 ± 0.32	1.47 ± 0.67 1.44 ± 0.73	5
Kim [89] 2013 Republic of Korea	Parallel groups 12-week follow-up	Age 53.6 ± 3.4 y, ysm 3.6 ± 2,4, BMI 23.3 ± 2.5, healthy	IC 70 mg (Glyc: 38 mg glycitin 20 mg daidzin, 12 mg genistin) vs. placebo	SG CG	42 43	5.13 ± 0.85 5.48 ± 1.03	2.97 ± 0.70 3.25 ± 0.92	1.48 ± 0.36 1.52 ± 0.37	1.26 ± 0.72 1.27 ± 0.66	4
Chilibec [83] 2013 Canada	Parallel groups 24-month follow-up	Age 56.6 ± 68 y, yms N/A, BMI 27.1 ± 4.1, healthy	IC 165 mg (150 mg Agl: Gen, Da and Gly in ratio of 1:1:0.5) vs. placebo	SG CG	72 73	5.87 ± 0.96 5.76 ± 0.91	3.68 ± 0.91 3.59 ± 0.89	1.58 ± 0.41 1.52 ± 0.44	1.41 ± 1.03 1.43 ± 0.79	4
Engelbert [86] 2016 Germany	Parallel groups 12-week follow-up	Age 59.5 ± 6.03 y, yms ≥ 1 y, BMI 25.2 ± 3.8, healthy	IAE 117.4 mg (49.7% Gen, 41.4% Dai, 9.0% Gly) vs. placebo, maltodextrin	SG CG	85 85	5.88 ± 0.89 5.80 ± 0.91	3.78 ± 0.89 3.67 ± 0.85	1.95 ± 0.44 1.99 ± 0.45	1.04 ± 0.39 1.04 ± 0.38	4
Barrasa [82] 2018 Chile	Parallel groups 1-week run-in 3-month follow-up	Age 64.7 ± 4.6 y, ysm N/A, BMI 27.6 ± 0.9, healthy	IAE 100 mg (52 mg Gen, 40 mg Dai, 8 mg Gly) vs. placebo	SG CG	20 15	5.13 ± 0.68 4.87 ± 0.62	3.10 ± 0.94 2.97 ± 0.50	1.30 ± 0.43 1.18 ± 0.38	1.53 ± 0.39 1.54 ± 0.36	4
**C. Red clover (*Trifolium pratense* L.)**										
Hale [96] 2001 Australia	Parallel groups 3-month follow-up	Age 47.2 ± 2.4 y, yms N/A, BMI 26.7 ± 4.6, healthy	IAE 50 mg (big amount of Bio and small amount of For (no data)) vs. placebo	RCG CG	14 14	4.64 ± 0.78 4.19 ± 0.85	2.89 ± 0.61 2.49 ± 0.73	1.29 ± 0.24 1.34 ± 0.43	1.46 ± 0.67 1.61 ± 1.04	4
Atkinson [94] 2004 United Kingdom	Parallel groups 12-month follow-up	Age 52.2 ± 4.8 y, yms N/A, BMI 25.3 ± 3.7, healthy	IAE 40 mg (24.5 mg Bio, 8.0 mg For, 1 mg Gen, 1 mg Dai) vs. placebo	RCG CG	77 86	6.34 ± 1.19 6.08 ± 1.04	4.21 ± 0.94 3.88 ± 1.00	1.61 ± 0.41 1.66 ± 0.48	1.24 ± 0.71 1.19 ± 0.66	3
Schult [100] 2004 USA	Parallel groups 2-week run-in 12-week follow-up	Age 52.3 ± 3.1 y, yms 3.2 ± 4.5, BMI 26.1 ± 4.9, healthy	IAE 82 mg (49 mg Bio, 14 mg For, 8 mg Gen, 7 mg Dai). IAE 57 mg (44.6 mg For, 5.8 mg Bio, 0.8 mg Dai, 0.8 mg Gly) vs. placebo	RCG 82 RCG 57 CG	81 81 83	5.76 ± 0.92 5.77 ± 1.01 5.72 ± 0.83	3.77 ± 1.01 3.81 ± 1.14 3.72 ± 0.79	1.36 ± 0.37 1.34 ± 0.34 1.38 ± 0.40	1.32 ± 0.65 1.31 ± 0.77 1.22 ± 0.56	4
Hilgado [97] 2005 Ecuador	Cross-over 90-day active phase 7-day washout	Age 51.3 ± 3.5 y, yms ≥ 1 y, BMI 26.1 ± 3.9, healthy	IAE 80 mg (49 mg Bio, 16 mg For, 8 mg Gen, 7 mg Dai) vs. placebo	RCG CG	53 53	5.79 ± 0.97 5.79 ± 0.97	3.80 ± 0.77 3.80 ± 0.77	1.03 ± 0.30 1.03 ± 0.30	2.28 ± 0.89 2.28 ± 0.89	4
Clifton-Bligh [95] 2015 Australia	Parallel groups 1-month run-in 12-month follow-up	Age 54.4 ± 3.9 y, yms ≥ 1 y, BMI 24.8 ± 4.3, healthy	IAE 57 mg (44.6 mg For, 5.8 mg Bio, 1.9 mg Dai, 0.8 mg Gen, 0.8 Gly) vs. placebo	RCG CG	56 47	5.91 ± 1.05 5.80 ± 0.88	3.68 ± 0.94 3.43 ± 0.86	1.67 ± 0.35 1.82 ± 0.49	1.33 ± 0.60 1.11 ± 0.63	5
Lambert [98] 2017 Denmark	Parallel groups 12-week follow-up	Age 52.5 ± 3.5 y, yms N/A, BMI 25.7 ± 4.3 healthy	IEA 33.8 mg (19 mg For, 9 mg Bio, 2.2 mg Gen, 1.6 Dai) vs. placebo	RCG CG	30 29	5.38 ± 0.19 5.63 ± 0.10	3.36 ± 0.16 3.40 ± 0.17	1.76 ± 0.15 1.73 ± 0.10	1.20 ± 0.09 1.18 ± 0.10	6
Lambert [99] 2017 Denmark	Parallel groups 12-month follow-up	Age 61.8 ± 6.4 y, amenorrhea ≥12 months, BMI 25.6 ± 4.5, healthy	IEA 55.8 mg (31.4 mg For, 14.9 mg Bio, 6.9 mg Gen, 2.6 mg Dai) vs. placebo	RCG CG	38 40	5.54 ± 0.86 5.64 ± 1.01	3.28 ± 0.86 3.37 ± 0.89	1.81 ± 0.43 1.82 ± 0.51	1.16 ± 0.37 1.38 ± 0.63	5

Data are presented as mean ± standard deviation (SD). Abbreviations: Agl, aglycone; ALA, α-linolenic acid; Bio, biochanin; BMI, body mass index (kg/m2); CG, control group; CP, casein protein; Dai, daidzein; FG, flaxseed group; For, formononetin; FXO, flaxseed oil; Gen, genistein; GFX, ground flaxseed; Gly, glycitein; Glyc, glycoside; HDL-C, high-density lipoprotein cholesterol; IAE; IC, isoflavone conjugate containing aglycone and glycoside; IF, isoflavones (form and composition unknown); LDL-C, low-density lipoprotein cholesterol; MP, milk protein; N/A, not available, RCG, red clover group; ref., reference; SDG, secoisolariciresinol diglucoside; SG, soy group; SP, soy protein; TAG, triacylglycerols; Total-C, total cholesterol; WFX, whole flaxseed; y, year or years; ysm, years since sine menopause.

## Data Availability

Not applicable.
